# Cost-utility analysis of ropeginterferon alfa-2b to manage low-risk patients with polycythemia vera as compared to phlebotomy only in the Austrian healthcare system

**DOI:** 10.1007/s00277-025-06229-w

**Published:** 2025-01-31

**Authors:** Evelyn Walter, Francesca Torelli, Tiziano Barbui

**Affiliations:** 1 IPF Institute for Pharmaeconomic Research, Vienna, Austria; 2 AOP Orphan Pharmaceuticals, Vienna, Austria; 3 FROM, Fondazione per la Ricerca Ospedale di Bergamo, Bergamo, Italy

**Keywords:** Myeloproliferative neoplasm, Polycythemia vera, Low-risk, Ropeginterferon alfa-2b, Phlebotomy, Cost-effectiveness

## Abstract

Treatment of polycythemia vera (PV) aims to maintain hematocrit on target to reduce risk of thrombotic complications, while preventing disease progression to myelofibrosis (MF) and acute myeloid leukemia (AML). This analysis evaluated cost-effectiveness of adding ropeginterferon alfa-2b (ropegIFNα) to phlebotomy in patients with low-risk PV (those younger than 60 years without prior thrombosis), compared to phlebotomy alone. We combined a 12-month decision tree with a semi-Markov cohort model comparing ropegIFNα to the standard treatment from the Austrian healthcare system perspective over 30 years. Outcomes were quality adjusted life years (QALYs), costs, and incremental cost-utility ratio (ICUR). Model inputs were obtained from the phase 2 Low-PV study, additional published literature and from Austrian-specific cost databases. One-way and probabilistic sensitivity analyses (SA) assessed the robustness of findings. RopegIFNα led to 1,43 higher QALYs and 50.960 EUR overall higher costs compared to phlebotomy alone, with an ICUR of 35.525 EUR/QALY. Thrombosis, MF, and AML costs decreased for the ropegIFNα group by 12%, 30% and 16% respectively, due to the delayed complications onset and disease progression. In the one-way SA, ropegIFNα costs and discount rates had the greatest impact on results. The probabilistic SA showed a 100% probability of cost-effectiveness at willingness-to-pay threshold aligned to the Austrian GDP per capita. RopegIFNα is a cost-effective treatment option for patients with low-risk PV. These findings suggest that early treatment with ropegIFNα could ensure optimal resource allocation by preventing costly thrombotic events and progression to MF whilst increasing patient quality of life.

**Supplementary Information:**

The online version contains supplementary material available at 10.1007/s00277-025-06229-w.

## Introduction

Polycythemia Vera (PV) is a rare, chronic myeloproliferative neoplasm (MPN) characterized by an excessive production of erythrocytes [1], with an annual incidence in Europe ranging from 0,4 to 2,8 cases per 100.000 individuals [2] and a prevalence of approximately 30 per 100.000 [3], predominantly affecting individuals in late adulthood [4]. In Austria, this translates to approximately 2.700 patients. PV leads to a spectrum of potentially debilitating symptoms, including fatigue, pruritus, pain, primarily due to increased blood viscosity, significantly impacting patients’ quality of life [5, 6]. The uncontrolled erythrocytes production elevates the risk of thrombotic events, with a reported 3-year incidence rate between 6% and 17% [7]. Over time, PV may progress to secondary myelofibrosis (MF) or acute myeloid leukemia (AML) [8], contributing to an average 13 years reduction in life expectancy compared to the general population [9]. Patients with PV face increased healthcare use, including higher rates of hospitalizations, outpatient visits, and emergency visits, driven by PV symptoms and thrombotic complications [10–12]. Progression to post-PV MF or AML, with poorer prognoses than PV, significantly increases healthcare use and costs, especially for outpatient and inpatient care [10, 13, 14].

The classification of PV patients into high- or low-risk categories based on age and history of thrombosis informs prognosis and guides treatment strategies [15]. Low-risk patients are typically managed with low-dose aspirin and phlebotomy [15]. However, despite these interventions, the incidence of vascular events remains approximately twice as high as that of the general population, with an estimated rate of 2,5 per 100 patients annually [16]. Furthermore, phlebotomy can be burdensome and poorly tolerated by some patients, leading to side effects such as iron deficiency, hypotension, headaches, fatigue, syncope, weakness, and dizziness, along with work absence and daily life disruptions [17–19].

Ropeginterferon alfa-2b (ropegIFNα) (BESREMi^®^; AOP Orphan Pharmaceuticals GmbH, Vienna, Austria) is a new generation interferon-α specifically developed for the treatment of PV, administered with a ready-to-use injection pen and offering improved pharmacokinetic and pharmacodynamic properties compared to older interferons [20, 21]. RopegIFNα was assessed in low-risk PV patients in the phase II Low-PV trial (NCT030030025) [22, 23]. Due to overwhelming efficacy of ropegIFNα with standard treatment versus phlebotomy alone, recruitment was stopped early (*N* = 127). The primary endpoint (hematocrit [HCT] target maintenance) was achieved in 84% of ropegIFNα patients versus 60% in the standard arm (*p* = 0,008), with no significant safety difference. Authors concluded that supplementing phlebotomy with ropegIFNα was safe and effective for the continuous maintenance of HCT target values in patients with low-risk PV, provoking revision of international treatment guidelines to extend use of interferon alfa in this patient population [24–26]. The updated treatment algorithm for low-risk PV highlights the need to evaluate the economic viability of utilizing ropegIFNα in this patient population. Thus, building on the findings of the Low-PV study, we aimed to conduct a cost-utility analysis comparing (1) conventional therapy with phlebotomy and low-dose aspirin (100 mg daily, unless contraindicated), and (2) ropegIFNα administered subcutaneously every two weeks at a fixed dose of 100 µg, in addition to conventional therapy, in low-risk PV patients within the Austrian healthcare context.

## Methods

### Model design

A Microsoft Excel cost-utility model compared ropegIFNα + phlebotomy versus standard treatment with phlebotomy alone for a hypothetical cohort of 1000 patients from the Austrian healthcare payer perspective (direct medical costs only). The 30-year-time horizon reflected the chronic nature of PV, capturing lifetime costs and outcomes including complications and disease progression. The analysis adhered to ISPOR and Austrian modelling guidelines [27, 28].

**Fig. 1:**
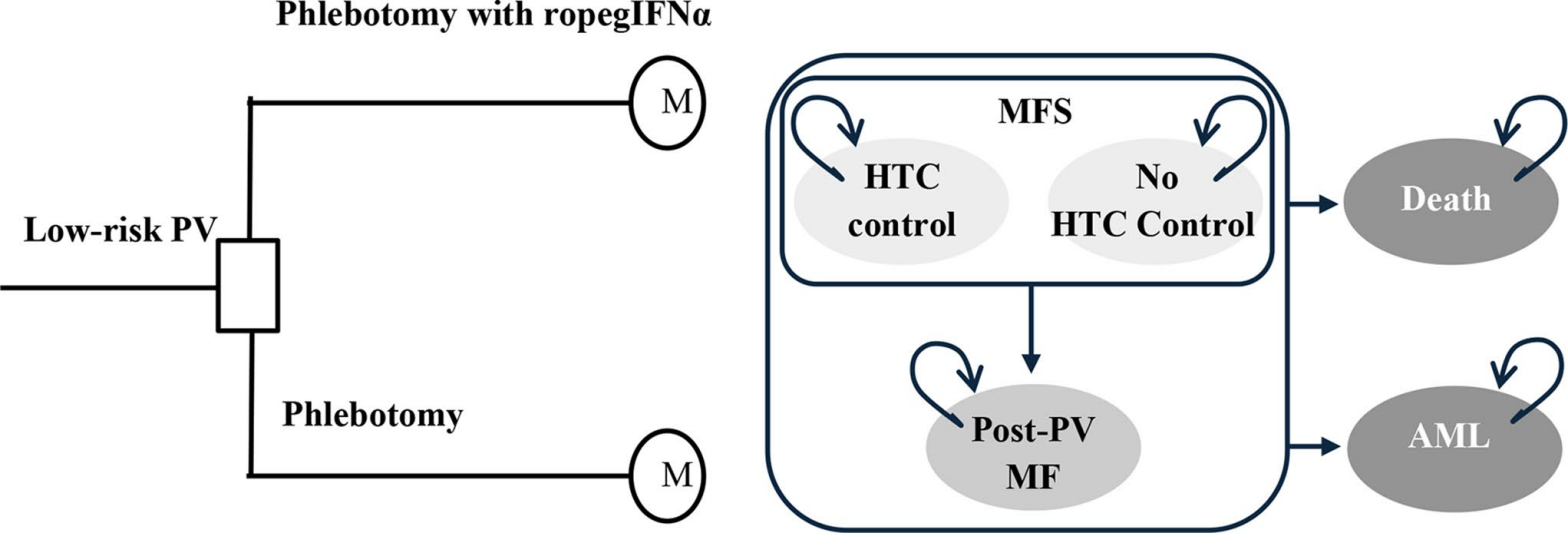
Model structure. AML, acute myeloid leukemia; HCT, hematocrit; MF, myelofibrosis; PV, polycythemia vera; ropegIFNα, ropeginterferon alfa-2b

The model used a 12-month decision tree (aligned with Low-PV study data) and a long-term Markov model to simulate a long-term follow-up (Fig. 1).

The decision tree’s endpoint was treatment response, defined as maintaining hematocrit (HCT) ≤ 45% without disease progression over 12 months. The following Markov model consisted of five health states: (1) MF-free survival (MFS) with HCT control (maintenance of median recommended value HCT ≤ 45%, WBC < 10 × 10^9^/L and PLT < 400 × 10^9^/L per cycle), (2) MFS without HCT control (maintenance of median recommended value HCT > 45%), (3) Post-PV MF (secondary MF with increased leukocytes (leukocytosis) and platelets (thrombocytosis) and splenomegaly), (4) AML, and (5) Death.

Patients transitioned between states every 3 months based on probabilities, with thrombotic risk higher in the no HCT control state. Each patient was assumed to receive a full course of treatment at the start of every 3-month cycle; therefore, a half-cycle correction was not required. Progression to post-PV MF or AML was modeled, and all states allowed transition to death. Key modelled complications were thromboembolic (TE) events (venous and/or arterial), bleeding/hemorrhage, post-PV MF, and AML transformation. TE events were not separate states but occurred based on HCT control state. The model calculated cumulative costs and quality adjusted life years (QALYs), discounted at 5% per year [28]. Deterministic one-way and probabilistic sensitivity analyses tested results robustness.

### Model inputs

#### Patients

The base-case population in the model followed the inclusion criteria of the Low-PV study. Patients entered the model at age 50, meeting the definition of low-risk PV according to the European LeukemiaNet and the National Comprehensive Cancer Network — i.e., they were younger than 60 years and had an absence of a history of arterial or venous thrombosis [23].

#### Clinical outcomes

The Low-PV study served as the primary source for clinical values in the 12-month decision tree (Supplementary Table 1). For the Markov model (including subsequent treatments), clinical values were derived from both the Low-PV study and additional published literature, specifically: hematological response data for ropegIFNα and hydroxyurea were sourced from the CONTINUATION-PV study [29] and data for ruxolitinib were obtained from the RESPONSE trial [30] (Supplementary Table 2).

#### Following treatments

In the Markov model, patients in the ropegIFNα group could switch therapy (interferons at higher dosage, hydroxyurea, or ruxolitinib) and those in the standard group could initiate cytoreductive treatment due to thrombosis, signaling a shift from low to high risk, or by suitability of hydroxyurea for patients reaching age 60. Time on treatment was initially explored using parametric survival curves (Exponential, Weibull, Gompertz, Log-normal, Log-logistic, Gamma) based on median durations from a large, single-center retrospective study [31] including 470 PV patients who received either recombinant interferon alpha-2a, hydroxyurea, or phlebotomy-only treatment. Among these options, the exponential model was chosen for its clinical plausibility.

#### Adverse events

While the adverse events (AEs) in the 12-month decision tree were sourced from the Low-PV study, different literature sources were used to record those of the following treatments (Supplementary Table 3). To capture the most clinically and economically relevant AEs, they were included within the economic model if they were grade 3/4 and occurred in 1% or more of patients receiving either ropegIFNα, phlebotomy, or follow-up therapy.

#### Complications and disease progression

History of thrombotic events was used as a prognostic factor of thrombotic and cardiovascular events. The annual rate of major arterial and venous events in low-risk PV patients is approximately 2% per patient-year [23, 32, 33]. To account for differences between patients achieving and not achieving the HCT target, a hazard ratio (HR) from Marchioli et al. (2013) was used. In that study, vein thrombosis occurred in 4,4% of the low-HCT group versus 10,9% of the high-HCT group (HR 2,69; 95% CI, 1,19–6,12; *p* = 0,02). The model assumes this HR applies to low-risk PV patients.

Progression to MF and AML was modelled based on Abu-Zeinah et al., 2021. MF progression was supplemented with data from a simulation multistate model [34] using data from a large series of 1.545 patients with PV.

#### Mortality

PV patients have higher mortality than the general population. For overall survival (OS), data from low-risk patients showed a 20-year OS of 87%, with rates of 100%, 85%, and 80% for recombinant interferon-alpha, hydroxyurea, and phlebotomy, respectively [31]. OS curves were extrapolated to 30 years using six parametric models (Exponential, Weibull, Gompertz, Log-normal, Log-logistic, Gamma), with the log-logistic model selected for clinical validity and best fit (AIC/BIC). Ruxolitinib’s OS curve was estimated as a weighted average of hydroxyurea and interferon due to missing data.

#### Utilities

Model utilities are listed in Supplementary Table 4. In the absence of PV-specific utility values, responders were assumed to have utility equal to the general population. As Austria-specific values were unavailable, base health state utilities from five European countries using EuroQol 5-Dimension were used [35]; a disutility of -0,117 was then applied for patients not achieving the HCT target and a further disutility of -0,152 was considered for the MF health state [36, 37]. For secondary AML, a disutility of -0,197 was applied [38, 39]. Thrombosis disutility was not included, being reflected in the utility difference between achieving and not achieving the HCT target. PV symptom frequencies for ropegIFNα and phlebotomy were modelled at baseline, 12 months, and 24 months using Low-PV data, with disutilities from the literature, and assumed to stabilize after year 2.

#### Healthcare resources and costs

Resource use (RU) and costs for each health state were based on the Low-PV study and published literature, focusing on direct medical costs from the Austrian payer’s perspective, adjusted to 2024 using the Austrian consumer price index [40]. Costs included treatments (drug acquisition and duration), subsequent therapies, monitoring, complications, AEs, and end-of-life care (Supplementary Table 4). Routine monitoring covered clinical exams, blood counts, and spleen sonography, with more frequent visits for patients failing to achieve the HCT goal and requiring shorter phlebotomy intervals. The total monitoring cost per cycle was calculated by multiplying each item (outpatient physician visit, blood count, and sonographic control of the spleen) by the corresponding resource use of patients with and without HCT control.

Costs for each complication were calculated as average of the inpatient and outpatient tariffs according to the expected setting of care. To ascertain the cost of thrombosis, the various thrombotic events [41] were recorded and evaluated in terms of cost. Austrian DRG codes were used to calculate average costs per thrombotic event.

The costs of post-PV MF were estimated through a micro-costing approach, since no study from Europe was found in a literature search whose costs could be reliable transferred to Austria. Real-world resource consumption [42] was linked to costs from Austria. From this information and additional resource consumption, costs of €31.557,19 per year were calculated (Supplementary Table 5).

Cost of AML was extracted from a UK cost-of-illness study [43], converted to euros and inflation-adjusted (May 2024 exchange rate £1 = 1.17 € and Consumer Price Index rising from 103.3 in 2012 to 146.9 in May 2024 derived from Statistik Austria). Costs per cycle of induction therapy, the costs per cycle of consolidation therapy, and the costs of allogeneic stem cell therapy (SCT) were included. Additionally, a Swedish study [44] documented that 17,5% of patients of comparable age underwent SCT. This resulted in a cost for AML of €86.853,17.

End-of-life costs included those incurred for the stays in intensive care and palliative care units, plus the costs of the corresponding DRG lump sum for the main diagnosis of PV, MF or AML.

### Sensitivity analyses

Deterministic sensitivity analysis used input ranges from 95% CIs or adjustments to baseline estimates (± 20% for costs, ± 10% for clinical data), shown in a tornado diagram [28]. Probabilistic sensitivity analysis employed Monte Carlo simulation (1.000 iterations), using gamma distributions for costs and beta distributions for probabilities/utilities. Results were visualized with a scatterplot [45] and cost-effectiveness acceptability curve (CEAC) [46].

## Results

### Base case analysis

RopegIFNα therapy generated 10,09 QALYs versus 8,65 QALYs for the standard group. The results showed that therapy with ropegIFNα is associated with a significant improvement in QALY, namely 1,43 QALYs or 17,2 months in perfect health compared to the standard group over the 30-year time horizon. The average total cost per patient was €269.883 in the ropegIFNα and €218.923 in the standard group from the perspective of the Austrian healthcare system, yielding a €50.960 cost difference. Drug costs accounted for the largest part of total costs: 72,4% in the ropegIFNα group and 55,0% in the standard group. However, from Year 8, the yearly drug costs of the standard group exceed the yearly drug costs of the ropegIFNα group. The analysis resulted in an incremental cost-utility ratio (ICUR) of €35.525 (Table 1).

**Table 1:** Base case results

Costs & Health Outcomes	RopegIFNα group	Standard group	Difference
Medication costs	195 262,08 €	120 446,19 €	74 815,89 €
Phlebotomy costs	1 463,66 €	1 562,96 €	-99,30 €
Monitoring costs	2 914,44 €	2 885,15 €	29,30 €
Thrombosis costs	1 012,86 €	1 147,52 €	-134,67 €
AE costs	1 968,30 €	1 028,74 €	939,56 €
Post-PV MF	53 621,83 €	76 586,94 €	-22 965,12 €
AML costs	9 127,01 €	10 847,78 €	-1 720,77 €
End-of-life costs	4 513,09 €	4 418,17 €	94,92 €
**Total costs**	269 883,26 €	218 923,45 €	50 959,82 €
**QALYs**	10,09	8,65	1,43
**ICUR**	35 524,78 €		

AE, adverse events; AML, acute myeloid leukemia; ICUR, incremental cost-utility ratio; MF, myelofibrosis; PV, polycythemia vera; QALY, quality adjusted life year; ropegIFNα, ropeginterferon alfa-2b

Since there is no defined willingness-to-pay (WTP) threshold in Austria, the GDP per capita of €52.372 was used instead (last available value of the Austrian National Bank for 2023 [47]). This resulted in a Net Monetary Benefit (NMB) of €24.167,11 (NMB = WTP * delta QALYs– delta Cost).

A higher percentage of low-risk PV patients treated with ropegIFNα on top of phlebotomy achieved the HCT target. After two years, in the ropegIFNα group, 63,9% of all patients reached the HCT target compared to 47,3% in the standard group; after 5 years, it was 52,7% vs. 40,0% and after 10 years 38,9% vs. 31,6%. In the ropegIFNα group, occurrence of thrombosis was delayed. After 2 years, 1,9% vs. 4,3% of patients had experienced thrombotic events in the ropegIFNα and phlebotomy group, with a smaller difference after 5 years (7,9% vs. 10,8% respectively). Over 1.000 patients, this translated to prevention of thrombotic events in 29 patients treated with ropegIFNα vs. phlebotomy only.

**Fig. 2:**
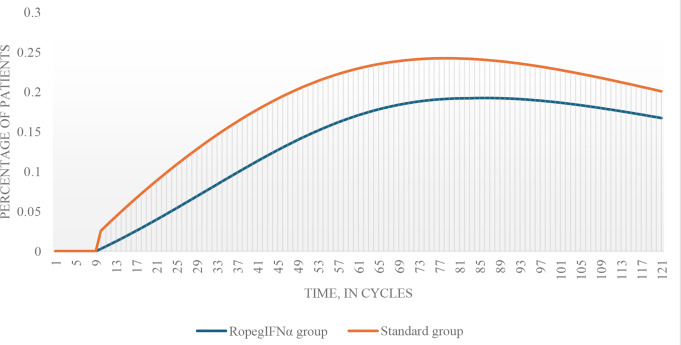
Simulated transition to post-PV MF. MF, myelofibrosis; PV, polycythemia vera; ropegIFNα, ropeginterferon alfa-2b

In our model, earlier use of ropegIFNα prevented the transition to post-PV MF. After 10 years, 11,3% of patients in the ropegIFNα group were predicted to progress to post-PV MF, compared to 17,8% in the phlebotomy group; after 30 years, 16,7% progressed in the ropegIFNα group compared to 20,0% in the phlebotomy group (Fig. 2).

**Fig. 3:**
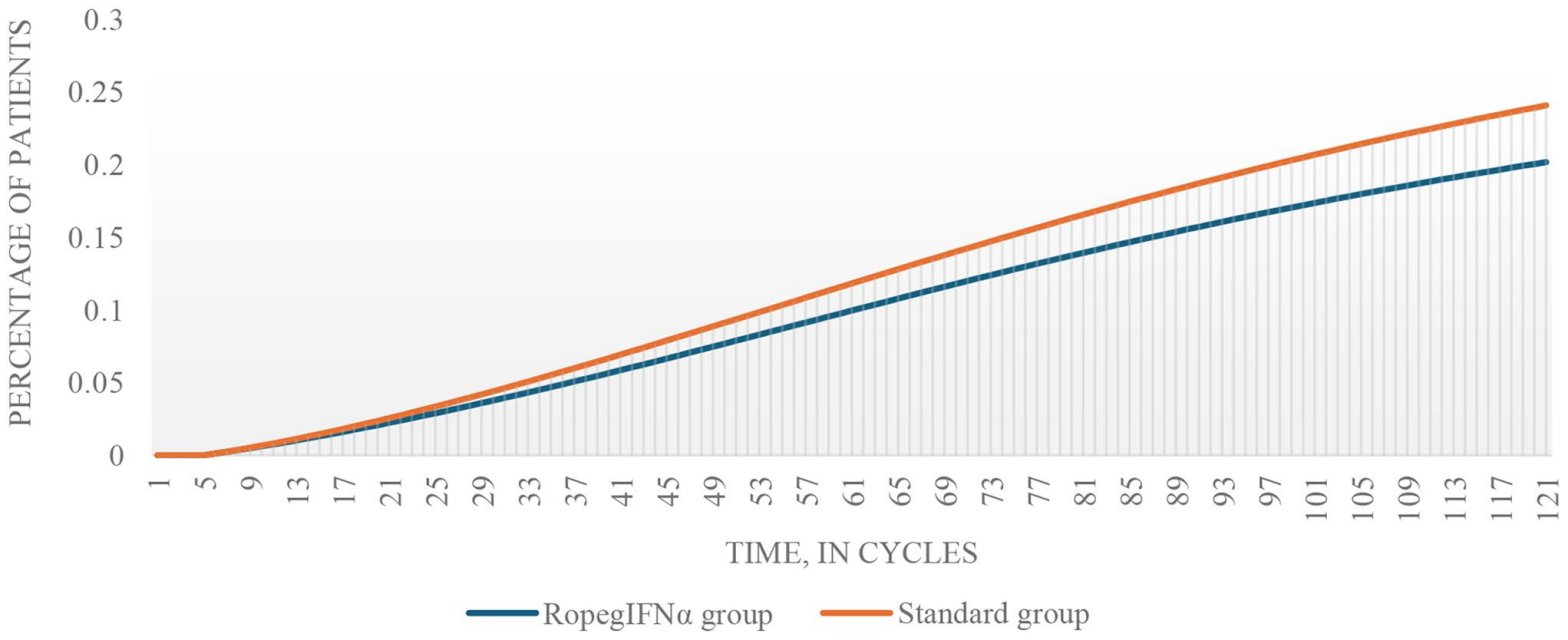
Simulated transition to AML. AML, acute myeloid leukemia; ropegIFNα, ropeginterferon alfa-2b.

Additionally, a higher percentage of simulated patients in the standard group developed AML (Fig. 3).

After 10 years, 6,9% of patients in this group were affected, compared to 5,9% in the ropegIFNα group, rising to 20,2% vs. 24,0% at 30 years. Over a cohort of 1.000 patients, this translated to 34 MF and 39 AML cases prevented over 30 years

### Sensitivity analyses results

**Fig. 4:**
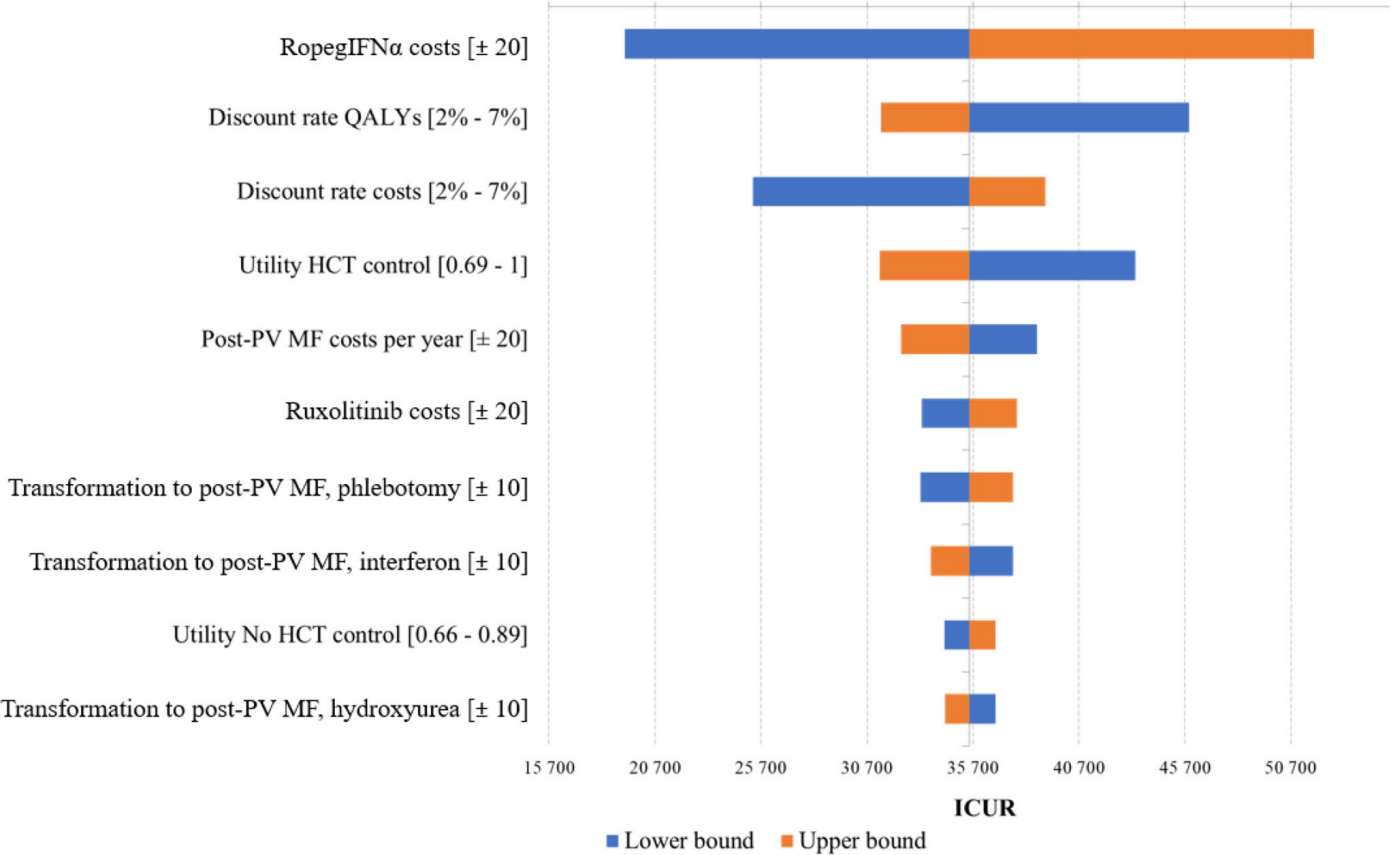
Tornado diagram. *HCT*,* hematocrit; ICUR*,* incremental cost-utility ratio; MF*,* myelofibrosis; PV*,* polycythemia vera; QALY*,* quality adjusted life year; ropegIFNα*,* ropeginterferon alfa-2b. Only the first 10 parameters with greater impact on the ICUR are listed*

One-way sensitivity analysis identified ropegIFNα costs, discount rates (QALYs and costs), and utility for HCT target as key drivers of uncertainty (Fig. 4).

**Fig. 5:**
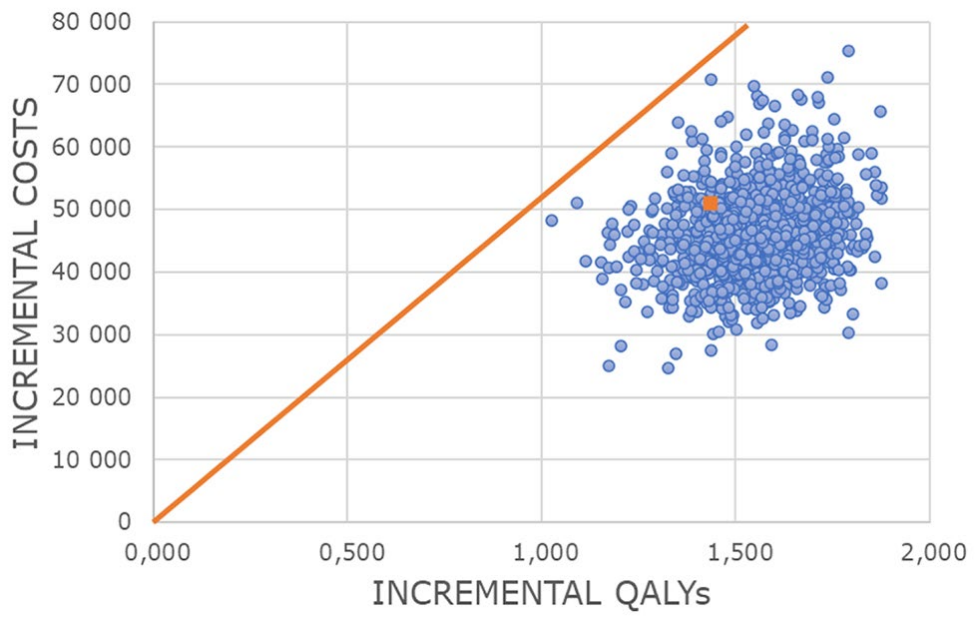
PSA scatterplot. *PSA*,* probabilistic sensitivity analysis; QALY*,* quality adjusted life year*

Costs of post-PV MF progression and ruxolitinib use also influenced results. In the PSA, 1,55 mean incremental QALYs were gained from ropegIFNα at a mean incremental cost of €45.866. The resulting probabilistic ICUR from 1.000 iterations was €29.562 (Fig. 5).

Probabilistic results aligned with the deterministic base case. RopegIFNα was cost-effective in around 100% of all simulations using the Austrian GDP (€52.372) [47] as WTP threshold.

## Discussion

In this 30-year cost-utility model, treatment with ropegIFNα contributed to better HCT control, slowed disease progression and increased quality of life of patients with low-risk PV compared to phlebotomy alone, whilst increasing overall costs. Results demonstrated cost-effectiveness of early treatment initiation with ropegIFNα, while patients are still in a disease stage with low risk of thromboembolic events, in the Austrian health care system. Sensitivity analyses confirmed robust results, with a 100% probability of cost-effectiveness at Austria’s GDP-based WTP threshold.

This study highlights the value of pharmacological treatment in low-risk PV patients over phlebotomy alone for maintaining HCT < 45% and reducing thrombotic risk. Improved QALYs with ropegIFNα stemmed from better HCT control and reduced symptoms like fatigue, itching, and abdominal discomfort. While initial drug costs were higher, by Year 8, yearly costs in the standard group surpassed those in the ropegIFNα group, reflecting the need for costlier treatments in patients without early ropegIFNα therapy.

Maintaining optimal HCT levels with phlebotomy alone is challenging and may increase thrombosis risk, as repeated phlebotomy can cause thrombocytosis. In the Low-PV study extension, 3,2% of phlebotomy-only patients developed thrombotic events, which were absent in the ropegIFNα group [22]. In our cohort model, unlike in the clinical trial, some patients switched to alternative therapies or discontinue treatment over time. As a result, our study predicted a 12% cost reduction with ropegIFNα due to fewer thrombotic complications. In the simulation, 64% of ropegIFNα patients achieved HCT control at 24 months, compared to 83% reported in the Low-PV study.

Phlebotomy does not prevent MF, while early ropegIFNα use delayed post-PV MF transition, reducing associated costs by 30%. Clinical studies link ropegIFNα to reduced JAK2V617F allele burden, a surrogate marker for disease modification and progression prevention [48]. In the Low-PV study, the 12-month mean JAK2V617F allele burden change was − 10,43% with ropegIFNα and 1,03% with standard treatment (*p* = 0.0060), with 16% of ropegIFNα patients showing a > 40% decrease [23]. Reducing JAK2V617F allele burden to prevent disease progression is crucial, as MF disrupts blood cell production, causing anemia, fatigue, and organ enlargement [49, 50], as well as decreased survival. Other complications associated with MF include portal hypertension, thromboembolism, and frequent infections [51, 52]. Moreover, progression to post-PV MF causes additional, avoidable resource use and costs related to outpatient and inpatient care [10] which can be prevented with early treatment optimization. In the Low-PV study, no patients on ropegIFNα experienced disease progression at 2 years, therefore our model allowed transitions to post-PV MF only after 24 months. However, 11% of simulated ropegIFNα patients progressed to post-PV MF over 10 years, likely overestimating progression compared to the 1% rate at Year 6 in the CONTINUATION-PV trial (vs. 3% in the hydroxyurea/BAT arm) [29]. This difference potentially occurred as in our cohort model, the disease progression rate in the ropegIFNα group was modelled with data from a large single center retrospective study of 470 PV patients with 10-year median follow-up [31], and represented a weighted average of different therapeutic approaches, with the proportion of patients on ropegIFNα decreasing over time due to treatment switching and discontinuation.

In PV, approximately 8–30% of patients with MF progress to blast-phase disease (AML); direct progression from PV to AML is instead less frequent (2%) [53]. In our model, a higher percentage of patients in the standard group developed AML, which is additionally connected to worsened clinical (including shorter life expectancy) and economic implications [13]. Although the difference in AML cases was small between the two groups, it yielded a 16% cost reduction for the ropegIFNα group due to the high cost of AML. Due to considering treatment switches and discontinuation over time, AML cases were also higher in our model compared to what has been observed in clinical trials, with no cases of AML in the ropegIFNα group (compared to 3% in the hydroxyurea/BAT arm) occurring in the CONTINUATION-PV study [29].

To our knowledge, this is the first analysis on cost-utility for ropegIFNα in Europe. The cost-effectiveness of ropegIFNα may vary across European countries due to differences in healthcare system structures, funding, and reimbursement policies. The findings, based on Austrian data, reflect the direct medical costs and healthcare resource utilization specific to Austria, which might not align with other European settings; however, the consistency in clinical benefits derived from clinical trials and in utilities sourced from five European countries supports the relevance of the findings for other countries, with appropriate adjustments. Economic studies in other European countries have shown that patients with PV experience more frequent hospitalizations, outpatient visits, and emergency room visits compared to controls, due to bothersome symptoms and thrombotic complications caused by uncontrolled disease. For example, a 2022 Danish registry-based, descriptive, matched cohort study [10] analyzed data for 1.109 PV patients from 2010 to 2016. In all years of follow-up, PV patients had a higher median number of GP contacts, inpatient days, outpatient visits, and treatments/examinations than their comparisons. A 2022 Spanish retrospective, multicenter study [54] analyzed hospital admission records of 490 PV patients registered between 2005 and 2019 and found an average admission cost of €5.580, increasing in patients deceased during the hospitalization. Hospital admissions were urgent or not scheduled in 75,0% of the cases, a percentage that increased with age. Further country-specific analyses are needed to validate the generalizability of findings to other healthcare systems. In the US, a cost-utility analysis of ropegIFNα used as first- or second- line treatment for treatment of both low-risk and high-risk PV patients (versus an alternative treatment pathway of first-line hydroxyurea followed by ruxolitinib) suggested that the benefits of treatment with ropegIFNα outweighed the costs for a broad range of patients with PV [36]. Over a lifetime, the model showed that patients who received ropegIFNα had higher QALYs (0,4), and higher cost ($60.175), with a cost per QALY of $141.783, confirming cost-effectiveness for a broad range of patients with PV, including both low- and high-risk patients and patients with and without prior cytoreductive treatment. Differently to the US analysis, our study is focused on low-risk PV patients only, starting treatment at 50 years; as reported in the ELN guidelines, ropegIFNα should be preferred for younger PV patients if not contraindicated, as the approved alternative (hydroxyurea) is linked to potential AEs (including infertility) and leukemia risk, as documented in longitudinal studies.

Data on the disutility of phlebotomy is lacking and could not be included in the analysis. While a key component of the current standard of care for PV, phlebotomy can be inconvenient and poorly tolerated by some patients. The frequent need for phlebotomy to maintain hematocrit levels below 45% poses significant challenges, often leading to non-compliance and clinical intolerance. Rapid fluid shifts can result in AEs such as hypotension, headaches, fatigue, syncope, weakness, and dizziness [17, 55]. Other side effects include dysphagia [55] and iron deficiency [18], which frequently contributes to fatigue and can potentially trigger reactive thrombocytosis. More than half of patients in the REVEAL study receiving phlebotomy experienced fatigue within 24 h after a phlebotomy, with bruising, dizziness, and dehydration being additional commonly reported effects [56]. Furthermore, one in five patients reported phlebotomies to be moderately or extremely bothersome and 16% reported that they were painful or physically uncomfortable. Among patients who experience repeat phlebotomies, poor venous access can become a challenge, and for patients with poor venous access at baseline, phlebotomy may not even be a viable option. In the Low-PV study, ropegIFNα patients required fewer phlebotomies, averaging 2,8 per patient versus 3,8 in the standard group (*p* = 0,029). Additionally, 16% of the 50 patients in the ropegIFNα group remained phlebotomy-free throughout the 12-month period [23].

Due to the perspective of the analysis, the indirect costs such as time and productivity loss are not accounted for when considering the frequency of phlebotomies. While specific data analyzing the societal burden of frequent phlebotomies is lacking, a Danish descriptive cohort study [57] analyzed labor market participation before and after diagnosis for 1.109 patients with PV vs. matched controls. Patients with PV were less likely to be working than comparators in the two years before diagnosis. From two years pre-diagnosis until two years post-diagnosis, the proportion of patients and comparators working consistently decreased while those receiving sickness benefits increased. Although in health economic evaluations, such allowances are considered transfer payments rather than indirect costs, these findings suggest a potentially significant socio-economic impact on personal income and productivity, as well as an increased reliance on social support systems.

The strength of our analysis lies in the utilization of robust clinical data from the Low-PV study to reflect clinically meaningful patient outcomes and treatment responses in the short term, and inclusion of additional literature to model a long-term horizon that captures the chronic nature of PV along with its associated costs and complications. The sensitivity analyses reinforce the results’ robustness, indicating a 100% probability of being cost-effective at a WTP threshold equivalent to the Austrian GDP per capita.

## Conclusions

RopegIFNα was cost-effective for low-risk PV patients in Austria, implying that its early adoption could help optimize longer term healthcare resource allocation. By preventing costly thrombotic events and delaying or halting progression to MF (and AML), the Austrian healthcare system can avoid the higher long-term costs associated with treating these severe complications. These findings support a policy argument for ensuring early access to ropegIFNα as a clinically effective and cost-effective intervention in the context of a resource-limited healthcare setting.

## Electronic supplementary material

Below is the link to the electronic supplementary material.

Supplementary Material 1

## Data Availability

The data that supports the findings of this study are available from the corresponding author upon reasonable request.
