# Supplementary material for: Cost-utility analysis of ropeginterferon alfa-2b to manage low-risk patients with polycythemia vera as compared to phlebotomy only in the Austrian healthcare system
Source: Ann Hematol. 2025 Jan 31;104(1):219–29. doi: 10.1007/s00277-025-06229-w (PMC11868240; doi:10.1007/s00277-025-06229-w)
Supplement: Supplementary file 1 — Supplementary Material 1 [file 277_2025_6229_MOESM1_ESM.docx]

**Supplementary Materials**

**Supplementary Table 1. Clinical data of the 12-month decision tree**

| Input | RopegIFNα group | DSA lower and upper values | Standard group | DSA lower and upper values | PSA distribution | *Source* |
| --- | --- | --- | --- | --- | --- | --- |
| Primary response | | | | | | |
| HCT control | 81.25% | 73.1 – 89.4% | 58.73% | 52.9 – 64.6% | Beta | Barbui, T. et al. (2023). *NEJM Evid*, *2*(6). |
| Phlebotomy free | 16.00% | 14.4 -17.3% | 0.00% | 0% - 10% | Beta | Barbui, T. et al. (2021). *Lancet Haematol*, *8*(3), e175-e184. |
| Disease progression | 0.00% | Not varied | 12.70% | Not varied | Beta | Barbui, T. et al. (2023). *NEJM Evid*, *2*(6). |
| Discontinuation of treatment | 0.00% | Not varied | 18.75% | Not varied | Beta | Barbui, T. et al. (2024). *Annals of Hematology*, *103*(2), 437-442. |
| Adverse events | | | | | | |
| Neutropenia | 8% | 7.1 – 8.7% | 0% | 0 – 0.1% | Beta | Barbui, T. et al. (2023). *NEJM Evid*, *2*(6); Barbui, T. et al. (2021). *Lancet Haematol*, *8*(3), e175-e184. |
| Pruritus | 2% | 1.8 – 2.2% | 0% | 0 – 0.1% | Beta | Barbui, T. et al. (2023). *NEJM Evid*, *2*(6); Barbui, T. et al. (2021). *Lancet Haematol*, *8*(3), e175-e184. |
| Hyper-transaminasemia | 2% | 1.8 - 2.2% | 0% | 0 – 0.1% | Beta | Barbui, T. et al. (2023). *NEJM Evid*, *2*(6); Barbui, T. et al. (2021). *Lancet Haematol*, *8*(3), e175-e184. |
| Skin symptoms | 0% | 0 – 0.1% | 4% | 3.7 – 4.5% | Beta | Barbui, T. et al. (2023). *NEJM Evid*, *2*(6); Barbui, T. et al. (2021). *Lancet Haematol*, *8*(3), e175-e184. |
| Pain not specified | 0% | 0 – 0.1% | 2% | 1.8% - 2.2% | Beta | Barbui, T. et al. (2023). *NEJM Evid*, *2*(6); Barbui, T. et al. (2021). *Lancet Haematol*, *8*(3), e175-e184. |
| Knee impingement syndrome | 0% | 0 – 0.1% | 2% | 1.8% - 2.2% | Beta | Barbui, T. et al. (2023). *NEJM Evid*, *2*(6); Barbui, T. et al. (2021). *Lancet Haematol*, *8*(3), e175-e184. |
| Thrombosis | 0% | 0 – 0.1% | 2% | 1.8% - 2.2% | Beta | Barbui, T. et al. (2023). *NEJM Evid*, *2*(6); Barbui, T. et al. (2021). *Lancet Haematol*, *8*(3), e175-e184. |

*DSA, deterministic sensitivity analysis; HCT, hematocrit; PSA, probabilistic sensitivity analysis; ropegIFNα, ropeginterferon alfa-2b.*

**Supplementary Table 2. Transition probabilities of the Markov model**

| Input | Base case value | DSA lower and upper values | PSA distribution | *Source* |
| --- | --- | --- | --- | --- |
| Initial treatments |  |  |  |  |
| HCT control change per cycle ropegIFNα group after month 12 | 1.17% | 1.05 – 1,29 | Beta | Gisslinger, H., et al. (2020). *Lancet Haematol*, *7*(3), e196-e208. |
| HCT control change per cycle standard group after month 12 | -2.18% | -1.96 –  -2,40 | Beta | Marchioli, R. et al. (2013). *N Engl J Med*, *368*(1), 22-33. |
| Following treatments | | | | |
| HCT control change per cycle 1st year with hydroxyurea | 17.20% | 15.5% - 18.9% | Beta | Gisslinger, H., et al. (2020). *Lancet Haematol*, *7*(3), e196-e208. |
| HCT control change per cycle 2^nd^ and 3^rd^ year with hydroxyurea | -2.73% | 2.45% - 3.00% | Beta | Gisslinger, H., et al. (2020). *Lancet Haematol*, *7*(3), e196-e208. |
| HCT control change per cycle after year 3 year with hydroxyurea | 0.00% | Not varied | Beta | Gisslinger, H., et al. (2020). *Lancet Haematol*, *7*(3), e196-e208. |
| HCT control change per cycle 1st year with interferon* | 13.49% | 12.14% - 14.84% | Beta | Gisslinger, H., et al. (2020). *Lancet Haematol*, *7*(3), e196-e208. |
| HCT control change per cycle 2^nd^ and 3^rd^ year with interferon* | 1.17% | 1.05 – 1,29 | Beta | Gisslinger, H., et al. (2020). *Lancet Haematol*, *7*(3), e196-e208. |
| HCT control change per cycle after year 3 year with interferon* | 1% | Not varied | Beta | Assumption |
| HCT control change per cycle 1st year with ruxolitinb | 11.66% | 10.49%-12.83% | Beta | Vannucchi, A. M. (2015). *Ann Oncol*, *26 Suppl 5*, v85-99. |
| HCT control change per cycle 2^nd^ and 3^rd^ year with ruxolitinb | 1.31% | Not varied | Beta | Vannucchi, A. M. (2015). *Ann Oncol*, *26 Suppl 5*, v85-99. |
| HCT control change per cycle after year 3 year with ruxolitinb | 1% | Not varied | Beta | Assumption |
| Rate of thrombosis |  |  |  |  |
| Thrombosis incidence per year | 2% | 1.8% - 2,2% | Beta | Barbui, T. et al. (2021). *Lancet Haematol*, *8*(3), e175-e184.; Barbui, T. et al. (2015). *American journal of hematology*, *90*(5), 434-437; Roncaglioni, M. C., et al. (2013). *N Engl J Med*, *368*(19), 1800-1808; Baigent, C. et al. (2009). *Lancet*, *373*(9678), 1849-1860. |
| HR HCT ≥ 0.45 | 2.69 | 1,19 – 6,12 | Beta | Marchioli, R. et al. (2013). *N Engl J Med*, *368*(1), 22-33. |
| Transformation to post-PV MF per cycle |  |  |  |  |
| Transformation rate with phlebotomy | 0.74% | 0.67% - 0.82% | Beta | Abu-Zeinah, et al. (2021). *Leukemia*, *35*(9), 2592-2601. |
| Transformation rate with interferon* | 0.22% | 0.20% - 0.24% | Beta | Abu-Zeinah, et al. (2021). *Leukemia*, *35*(9), 2592-2601. |
| Transformation rate with hydroxyurea | 0.54% | 0.48% - 0.59% | Beta | Abu-Zeinah, et al. (2021). *Leukemia*, *35*(9), 2592-2601. |
| Transformation rate with ruxolitinb | 0.33% | 0.29% - 0,36% | Beta | Barbui, T. et al. (2023). *NEJM Evid*, *2*(6). |
| Transformation to AML |  |  |  |  |
| Transformation rate with phlebotomy | 0.23% | 0.21% - 0.25% | Beta | Abu-Zeinah, et al. (2021). *Leukemia*, *35*(9), 2592-2601. |
| Transformation rate with interferon* | 0.11% | 0.10% - 0.21% | Beta | Abu-Zeinah, et al. (2021). *Leukemia*, *35*(9), 2592-2601. |
| Transformation rate with hydroxyurea | 0.19% | 0.17% - 0.21% | Beta | Abu-Zeinah, et al. (2021). *Leukemia*, *35*(9), 2592-2601. |
| Transformation rate with ruxolitinb | 0.14% | 0.13% - 0.16% | Beta | Vannucchi, A. M. (2015). *Ann Oncol*, *26 Suppl 5*, v85-99. |
| Transformation rate with post-PV MF | 0.41% | 0.26% - 0.45% | Beta | Yogarajah, M., & Tefferi, A. (2017, July). In *Mayo Clinic Proceedings* (Vol. 92, No. 7, pp. 1118-1128). Elsevier. |

*AML, acute myeloid leukemia; DSA, deterministic sensitivity analysis; HCT, hematocrit; HR, hazard ratio; MF, myelofibrosis; PV, polycythemia vera; ropegIFNα, ropeginterferon alfa-2b*.*Recombinant interferon alpha-2a, recombinant interferon alpha-2b, and pegylated interferon alpha-2a

The time adjustment is made with the following formula:

$$=1-EXP(-(-LN(1-"Percentage to be converted from")/("Time to be converted from"/"Time to be converted to")))$$

**Supplementary Table 3. Adverse events of following treatments (yearly incidence)**

| **Adverse events** | **Interferon** | **Hydroxyurea** | **Ruxolitinib** |
| --- | --- | --- | --- |
| Anemia | 0.23% | 0.45% | 1.20% |
| Thrombocytopenia | 0.68% | 1.14% | 2.40% |
| Leukopenia | 0.68% | 1.37% | 0% |
| Diarrhea | 0% | 0% | 1.20% |
| Abdominal pain | 0% | 0% | 0% |
| Fatigue | 0% | 0% | 1.20% |
| Asthenia | 0% | 0% | 0% |
| Arthralgia | 0.23% | 0% | 1.20% |
| Headache | 0% | 0% | 0.60% |
| Pruritus | 0% | 0% | 0% |
| Hypertransaminasemia | 2.31% | 1.14% | 0% |
| Alanine aminotransferase increased | 1.14% | 0% | 0% |
| Hypertension | 0.91% | 1.14% | 0% |
| Aspartate aminotransferase increased | 0.68% | 0% | 0% |
| Influenza | 0.45% | 0.45% | 0% |
| Neutropenia | 0.45% | 0.45% | 0% |
| Skin symptoms | 0% | 0% | 0% |

**Supplementary Table 4.** Input parameters; utility and costs

| Input | Base case value |  | PSA distribution | Source |
| --- | --- | --- | --- | --- |
| Utilities |  |  |  |  |
| HCT control utility | 0,861 | 0.69 - 1 | Beta | Janssen, M. F., et al. (2021). *Eur J Health Econ*, *22*(9), 1467-1475; Gerds, A. T., et al. (2023). *J Comp Eff Res*, *12*(9), e230066. |
| No HCT control | 0,744 | 0.60 – 0.89 | Beta | Janssen, M. F., et al. (2021). *Eur J Health Econ*, *22*(9), 1467-1475; Gerds, A. T., et al. (2023). *J Comp Eff Res*, *12*(9), e230066. |
| Disutilities |  |  |  |  |
| Post-PV MF | 0,152 | 0.18 – 0.12 | Beta | Gerds, A. T., et al. (2023). *J Comp Eff Res*, *12*(9), e230066. |
| AML | 0,197 |  | Beta | Tolley, K., et al. (2010). *J Med Econ*, *13*(3), 559-570. |
| Fatigue | 0,085 | 0.07 – 0.10 | Beta | *NICE TA722 (Table 45 CS129)* |
| Early satiety | 0,075 | 0.06 – 0,09 | Beta | *NICE TA356 [ID5106]* |
| Abdominal discomfort | 0,075 | 0.06 - 0.09 | Beta | *NICE TA356 [ID5106]* |
| Inactivity | 0,075 | 0.06 – 0,09 | Beta | *NICE TA356 [ID5106]* |
| Concentration problems | 0,075 | 0.06 – 0,09 | Beta | *NICE TA356 [ID5106]* |
| Night sweats | 0,075 | 0.06 – 0,09 | Beta | *NICE TA356 [ID5106]* |
| Itching | 0,075 | 0.06 – 0,09 | Beta | *NICE TA356 [ID5106]* |
| Bone pain | 0,075 | 0.06 – 0,09 | Beta | *NICE TA356 [ID5106]* |
| Fever | 0,075 | 0.07 – 0.11 | Beta | *NICE TA356 [ID5106]* |
| Weight loss | 0,090 | 0.06 – 0,09 | Beta | *NICE TA356 [ID5106]* |
| Costs |  |  |  |  |
| Phlebotomy | 37,38 € | 29.90 € - 44.86€ | Gamma | LKF Modell 2024 [Austrian DRG-System], Tariff catalogues of the 9 ÖGKs (weighted population average) |
| Monitoring costs for patients with HCT control (per cycle) | 41,74 € | 33.39 € - 50.09 € | Gamma | Tariff catalogues of the 9 ÖGKs (weighted population average) |
| Monitoring costs for patients without HCT control (per cycle) | 83,48 € | 66.78 € - 100.18 € | Gamma | Tariff catalogues of the 9 ÖGKs (weighted population average) |
| Thrombosis (event) | 3 593,80 € | 2,875.04 € 4,312.56 € | Gamma | Leistungsorientierte Krankenanstaltenfinanzierung (LKF) Modell 2024 |
| Post-PV MF (annual) | 31 557,19 € | 25,245,75 € - 37,868.62 € | Gamma | Microcosting based on Passamonti et al. 2022 |
| AML (annual) | 86 853,17 € | 69,482,54 € - 104,223.80 € | Gamma | Zeidan, A. M. et al. (2016). *Expert Rev Hematol*, *9*(1), 79-89. |
| Neutropenia | 3 130,79 € | 2,504.63 € - 3,756.95 € | Gamma | LKF Modell 2024, Tariff catalogues of the 9 ÖGKs (weighted population average) |
| Pruritus | 1 219,07 € | 975.26 € - 1,462.88 € | Gamma | LKF Modell 2024, Tariff catalogues of the 9 ÖGKs (weighted population average) |
| Hypertransaminasemia | 835,53 € | 668.42 € - 1,002.63 € | Gamma | LKF Modell 2024, Tariff catalogues of the 9 ÖGKs (weighted population average) |
| Flu-like symptoms | 1 424,89 € | 1,139.91 € - 1,709.87 € | Gamma | LKF Modell 2024, Tariff catalogues of the 9 ÖGKs (weighted population average) |
| Astenia | 1 221,64 € | 977.31 € - 1,465.97 € | Gamma | LKF Modell 2024, Tariff catalogues of the 9 ÖGKs (weighted population average) |
| Skin symptoms | 1 219,07 € | 975.26 € - 1,462.88 € | Gamma | LKF Modell 2024, Tariff catalogues of the 9 ÖGKs (weighted population average) |
| Pain not specified | 773,32 € | 618.65 € - 927.98 € | Gamma | LKF Modell 2024, Tariff catalogues of the 9 ÖGKs (weighted population average) |
| Knee impingement syndrome | 1 008,85 | 807,08 - 1 210,62 | Gamma | LKF Modell 2024, Tariff catalogues of the 9 ÖGKs (weighted population average) |
| Anaemia | 1,154.95 € | 923.96 € - 1,385.94 € | Gamma | LKF Modell 2024, Tariff catalogues of the 9 ÖGKs (weighted population average) |
| Thrombocytopenia | 1,095.38 € | 876.30 € - 1,314.46 € | Gamma | LKF Modell 2024, Tariff catalogues of the 9 ÖGKs (weighted population average) |
| Diarrhea | 599.09 € | 479.27 € - 718.91 € | Gamma | LKF Modell 2024, Tariff catalogues of the 9 ÖGKs (weighted population average) |
| Abdominal pain | 620.12 € | 496.10 € - 744.14 € | Gamma | LKF Modell 2024, Tariff catalogues of the 9 ÖGKs (weighted population average) |
| Fatigue | 1,221.68 € | 977.34 € - 1,466.02 € | Gamma | LKF Modell 2024, Tariff catalogues of the 9 ÖGKs (weighted population average) |
| Arthralgia | 2,114.88 € | 1,691.90 € - 2,537.86 € | Gamma | LKF Modell 2024, Tariff catalogues of the 9 ÖGKs (weighted population average) |
| Headache | 758.22 € | 606.58 € - 909.86 € | Gamma | LKF Modell 2024, Tariff catalogues of the 9 ÖGKs (weighted population average) |
| Alanine aminotransferase increased | 1,633.50 € | 1,306.80 € - 1,960.20 € | Gamma | LKF Modell 2024, Tariff catalogues of the 9 ÖGKs (weighted population average) |
| Hypertension | 767.52 € | 614.02 € - 921.02 € | Gamma | LKF Modell 2024, Tariff catalogues of the 9 ÖGKs (weighted population average) |
| Aspartate aminotransferase increased | 1,633.50 € | 1,306.80 € - 1,960.20 € | Gamma | LKF Modell 2024, Tariff catalogues of the 9 ÖGKs (weighted population average) |
| End of life | 16 306,95 | 13,045.56 € - 19,568.34 € | Gamma | Überregionale Auswertung der österr. Krankenanstalten |

AML, acute myeloid leukemia; HCT, hematocrit; MF, myelofibrosis; PV, polycythemia vera.

**Supplementary Table 5.** MF micro-costing components

| **Resource use** | **in %** | **Costs in €** | **Source** |
| --- | --- | --- | --- |
| Treatment alternatives |  |  |  |
| JAK2 inhibitors | 37.5% | 62,157.87 | Passamonti et al. (2022). *Future Oncology*, *18*(18), 2217-2231; Mehta et al. (2014). *Leukemia & Lymphoma*, *55*(10), 2368-2374.; Landesgesetzblatt für Wien (Wiener Selbstzahler) 2023 |
| Hydroxyurea | 39.2% | 792.05 | Passamonti et al. (2022). *Future Oncology*, *18*(18), 2217-2231; Mehta et al. (2014). *Leukemia & Lymphoma*, *55*(10), 2368-2374.; Landesgesetzblatt für Wien (Wiener Selbstzahler) 2023 |
| Prednisolone | 6.6% | 120.00 | Passamonti et al. (2022). *Future Oncology*, *18*(18), 2217-2231; Mehta et al. (2014). *Leukemia & Lymphoma*, *55*(10), 2368-2374.; Landesgesetzblatt für Wien (Wiener Selbstzahler) 2023 |
| Erythropoietin | 5.7% | 21,102.38 | Passamonti et al. (2022). *Future Oncology*, *18*(18), 2217-2231; Mehta et al. (2014). *Leukemia & Lymphoma*, *55*(10), 2368-2374.; Landesgesetzblatt für Wien (Wiener Selbstzahler) 2023 |
| Erythrocyte transfusion | 17.4% | 15,990.52 | Passamonti et al. (2022). *Future Oncology*, *18*(18), 2217-2231; Mehta et al. (2014). *Leukemia & Lymphoma*, *55*(10), 2368-2374.; Tariff catalogues of the 9 ÖGKs (weighted population average) |
| Second-line lenalidomide | 1.1% | 27,298.74 | Passamonti et al. (2022). *Future Oncology*, *18*(18), 2217-2231; Mehta et al. (2014). *Leukemia & Lymphoma*, *55*(10), 2368-2374.; Landesgesetzblatt für Wien (Wiener Selbstzahler) 2023 |
| Second-line interferon | 0.9% | 8,457.57 | Passamonti et al. (2022). *Future Oncology*, *18*(18), 2217-2231; Mehta et al. (2014). *Leukemia & Lymphoma*, *55*(10), 2368-2374.; Landesgesetzblatt für Wien (Wiener Selbstzahler) 2023 |
| Outpatient office visits | 98.8% | 844.20 | Österreichischer Apothekerverlag, Warenverzeichnis May 2024; Leistungsorientierte Krankenanstaltenfinanzierung (LKF) Modell 2024 |
| Monitoring | 100.0% | 500.88 | LKF Modell 2024 |
| Hospitalization | 34.0% | 6,032.44 | Österreichischer Apothekerverlag, Warenverzeichnis May 2024;  Bundesministerium Soziales, Gesundheit, Pflege und Konsumentenschutz 2023 |
| Emergency care | 42.0% | 415.12 | Österreichischer Apothekerverlag, Warenverzeichnis May 2024 |
| Total cost of MF |  | 31,557.19 |  |

LKF, Leistungsorientierte Krankenanstaltenfinanzierung; MF, myelofibrosis.

**Supplementary Figure 1.** CEAC for PSA at a willingness-to-pay corresponding to the Austrian GDP (€52.372)

PSA, probabilistic sensitivity analysis.
